# 3D Vision by Using Calibration Pattern with Inertial Sensor and RBF Neural Networks

**DOI:** 10.3390/s90604572

**Published:** 2009-06-11

**Authors:** Erkan Beṣdok

**Affiliations:** Erciyes University, Engineering Faculty, Geomatics Eng. Dept, 38039, Kayseri, Turkey E-Mail: ebesdok@erciyes.edu.tr

**Keywords:** camera calibration, MDLT, differential evolution, RBF neural networks

## Abstract

Camera calibration is a crucial prerequisite for the retrieval of metric information from images. The problem of camera calibration is the computation of camera intrinsic parameters (i.e., *coefficients of geometric distortions, principle distance* and *principle point*) and extrinsic parameters (i.e., 3D spatial orientations: *ω, ϕ, κ*, and 3D spatial translations: *t_x_, t_y_, t_z_*). The intrinsic camera calibration (i.e., *interior orientation*) models the imaging system of camera optics, while the extrinsic camera calibration (i.e., *exterior orientation*) indicates the translation and the orientation of the camera with respect to the global coordinate system. Traditional camera calibration techniques require a predefined mathematical-camera model and they use prior knowledge of many parameters. Definition of a realistic camera model is quite difficult and computation of camera calibration parameters are error-prone. In this paper, a novel *implicit* camera calibration method based on Radial Basis Functions Neural Networks is proposed. The proposed method requires neither an exactly defined camera model nor any prior knowledge about the imaging-setup or classical camera calibration parameters. The proposed method uses a calibration grid-pattern rotated around a *static*-fixed axis. The rotations of the calibration grid-pattern have been acquired by using an Xsens MTi-9 inertial sensor and in order to evaluate the success of the proposed method, 3D reconstruction performance of the proposed method has been compared with the performance of a traditional camera calibration method, Modified Direct Linear Transformation (MDLT). Extensive simulation results show that the proposed method achieves a better performance than MDLT aspect of 3D reconstruction.

## 1. Introduction

Camera calibration is a major issue in computer vision since it is related to many vision problems such as *neurovision, remote sensing, photogrammetry, visual odometry, medical imaging*, and *shape from motion/silhouette/shading/stereo*. Metric information within images can be supplied only by the calibrated cameras [1, 2]. The 3D computer vision problem is mathematically determined only if the optical parameters (i.e., *parameters of intrinsic orientation*) and geometrical parameters (i.e., *parameters of extrinsic orientation*) of the camera system are precisely known. The camera calibration methods can be classified according to the determination methods of *optical* and *geometrical* parameters of the imaging system [1]. The number of camera calibration parameters (i.e., *rotation angles, translations, coordinates of principal points, scale factors, skewness between image axes, radial lens distortion coefficients, affine-image parameters*, and *lens-decentering parameters*) depends on the mathematical model of the camera used [2].

In the literature, many camera calibration methods have been introduced. A self-calibration method to estimate the *optic* and *geometric* parameters of a camera from vertical line segments of the same height is examined in [3]. Extrinsic calibration of multiple cameras is very important for 3D metric information extraction from images. Computation of relative orientation parameters between multiple photo/video cameras is still one of the active research fields in the computational vision [4, 5]. Using geometric constraints within the images, such as lines and angles, enables performing 3D scene reconstruction tasks with fewer images [6].

Plane-based camera calibration is an active area in computational vision because of its flexibility [7]. A planar calibration grid-pattern has some important advantages with respect to 3D calibration objects such as simple design, simple structure, easy scaling and easy construction. Therefore, planar calibration objects are preferred in computer vision applications [8]. Planar calibration objects and projective constraints can be used for calibration of parametric and nonparametric distortions of a camera system [9]. The camera calibration problem for planar robotic manipulators through visual servoing under a fixed-camera configuration has been investigated in [10].

Dual images of spheres and the dual image of the absolute conic have been used for solving the problem of camera calibration from spheres in [11]. The mirror-symmetric objects have been used for camera calibration in [12]. An accurate calibration procedure has been introduced for fish-eye lenses in [13]. The calibration of a projector-camera system by estimating the homography has been investigated in [14]. Online calibration methods have been used in virtual reality applications in [15]. A dynamic calibration method for multiple cameras has been investigated in [16]. Due to the noise-influenced image coordinates, most of the existing camera calibration techniques are unsuccessful aspects of robustness and accuracy.

The artificial neural networks (ANNs) can mimic the transformation between the image plane and the global coordinate system. By using ANNs, it becomes unnecessary to know both the physical parameters and the geometrical parameters of the imaging systems for 3D perception of objects from their 2D images. ANNs have been intensively used for camera calibration in some recently introduced methods [17, 18, 19]. A planar pattern has been observed at different rotations for setting training and test data sets of the ANN used. The rotation value of the planar pattern has been acquired by using an Xsens MTi-9 inertial sensor [20, 21]. With the proposed method, the 3D global coordinates of object points have been predicted from their 2D corresponding image coordinates.

The Xsens MTi-9 sensor is a miniaturized, gyro-based Attitude and Heading Reference System whose internal signal processor provides drift-error free 3D acceleration, 3D orientation, and 3D earth-magnetic field data. The drift-error growing nature of inertial systems limits the accuracy of inertial measurement devices. Inertial sensors can supply reliable measurements only for small time intervals. The inertial sensors have been used in some recent research for stabilization and control of digital cameras, calibration patterns and other equipment [22, 23].

The Modified Direct Linear Transformation (MDLT) is one of the commonly used camera calibration methods in computational vision applications for 2D and 3D object reconstruction [24]. The success of the proposed method has been evaluated by comparing the test results of the proposed method and MDLT method.

The camera calibration methods have been classified into two main classes in the literature: *explicit* and *implicit* camera calibration methods. The *explicit camera calibration* means the process of computing the physical parameters of a camera. The proposed method is classified as an implicit camera calibration method and implicit camera calibration methods do not require physical parameters of cameras for back-projection.

The rest of the paper is organized as follows: *Artificial Neural Networks* are explained in Section 2. *Proposed Method* and *Experiments* are given in Section 3 and Section 4, respectively. Finally, *Results and Discussion* are given in Section 5.

## 2. Artificial Neural Networks (ANNs)

An ANN is a network of neurons, which mimics a biological information processing system [25]. ANNs have been used to solve some of the complex problems in the fields of multicamera calibration, modeling of geometric distortions of image-sensors, stereo-vision, image denoising, image enhancement, and image restoration. In this paper, ANNs are applied to nonlinear problem of multicamera calibration for 3D information extraction from images. Camera calibration is an unavoidable-step for extraction of precise 3D metric information from images. In recent years some hybrid camera calibration techniques based on ANNs have been proposed for back-projection or 3D reconstruction without using a predefined camera model [17, 18, 19].

In this paper, a *Radial Basis Function Based Artificial Neural Network* (RBF) [26] is used to calibrate a multicamera system. A four-input and three-output architecture of RBF has been adopted to transform the image coordinates to their corresponding 3D spatial coordinates.

### 2.1. Training of Radial Basis Function Neural Networks

RBF has been successfully applied to many scientific research areas including *image enhancement, surface reconstruction, classification*, and *computational vision*. In order to use an RBF, the training functions of the hidden-layer and output-layer, the number of neurons in the related layers, and a performance measure for modeling the quality of learning phase must be specified. The computation phase of the RBF weights is called *network training*. In the last decade several methods were introduced in the literature for training RBFs [27, 28]. RBF has a three-layered ANN architecture: An input layer, a hidden layer and an output layer.

The RBF with Gaussian functions is defined as in [27];

(1) $$\begin{array}{cc} \delta_{i}(\lambda )=\sum_{ɛ=1}^{N}w_{i,ɛ}e^{-\frac{{\left\|\lambda -c_{ɛ}\right\|}^{2}}{2\sigma_{ɛ}^{2}}}, & \;i=1,2,3,\ldots ,I \end{array}$$

where

‖…‖ : Euclidean norm,
*c_ε_* : The center,
*σ_ε_* : The width of the *ε^th^* neuron in the hidden layer,
*w_i,ε_* : The weights in the output layer,
*N* : The number of Gaussian neurons in the hidden layer,
*λ* : Input pattern of RBF,
*δ* : Output pattern of RBF,
*I* : The number of neurons in the output layer.

The Root-Mean-Squared-Error (RMS), Mean-Squared-Error (MSE), Sum-Squared-Error (SSE), and Mean-Absolute-Error (MAE) functions have been examined as fitness functions. The influence of fitness function on the architectural structure of RBF has been analyzed and the results have been tabulated in Table 1.

In this paper, the RMS has been used as fitness function and it is formulated as;

(2) $$\text{RMS}=\sqrt{\frac{1}{N_{t}}\sum_{n=1}^{N_{t}}{\left(p_{n}-y_{n}\right)}^{2}}$$

where *p_n_* and *y_n_* denote the *desired output* and the *network output* for pattern *n*, respectively. *N_t_* is the number of training patterns.

The value of *N* is very important since it affects the *generalizing ability, architectural structure* and *computational-burden* of the RBF. Insufficient value of *N* leads to a poor learning performance. Infinite number of basis functions reduce the value of fitness function to zero but this causes *overlearning* [25], therefore the predefined rule of *N* ≤ 50 has been used as a condition of minimizing the *fitness function.* Design of the RBF requires the detection of optimum values of *N,w_i,ε_, σ_ε_* and *c_ε_*. The training phase has been stopped when the value of *fitness function* ≤ 0.01 has been reached.

In this paper, the RBF has been trained by using Differential Evolution algorithm (DE). DE [29] is a class of genetic-algorithm based search technique, which has robust search ability and it can be used to train ANNs including RBF. The main advantages of DE can be summarized as; easy implementation, fast convergence, limited number of control parameters, and finding the global minimum regardless of the high-quality values of initial parameters.

## 3. Proposed Method

**Figure 1.**
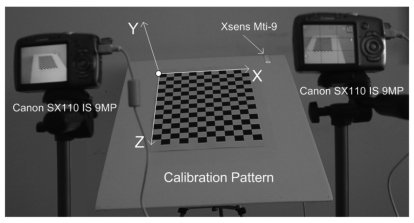
Experimental Setup and Rotating-Calibration Pattern.

In this paper, a stereo vision system has been calibrated by using the proposed method. The proposed method uses a 2D planar grid-pattern object (Figure 1), in order to perform the calibration process. The application steps of the proposed method are explained below.

**Figure 2.**
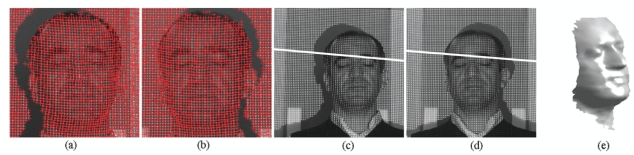
Experiments using the author's face: (a)-(b)Harris points on stereo images, (c)-(d)The stereo images with an epipolar line, (e) 3D solid mesh model of the author's face.

1. *Training Data Arrangement for RBF*: In this step, the calibration grid-pattern has been arbitrarily rotated towards the cameras around the *static*-fixed axis in *five* approximately equal steps and the stereo images have been captured with two static cameras at the end of each rotation. There are 219 grid-corners on the calibration pattern and totally 1,095 grid-corners have been observed. The number of randomly selected observations of grid-corners have been used as 795 (which is the value of *N_t_* in Equation 2) for the *training set* of RBF and the remaining 300 observations of grid-corners have been used for the generalization *test set* of RBF.
  In the case of rotating the calibration plane arbitrarily without a fixed axis in calibration space, both the *spatial translations* and *spatial rotations* must be observed, in order to compute the exact position of 3D grid corners on calibration pattern in 3D space. In order to avoid additional observation parameters (i.e., *spatial translations*), a fixed axis has been used in this paper.
  The rotation matrix of calibration grid-pattern has been acquired at 100 Hz by using an Xsens MTi-9 sensor attached to calibration pattern. The *object reset* function of the SDK of Xsens aims to facilitate aligning the MTi-9 sensor coordinate frame with the 3D global coordinate frame of the calibration grid-pattern to which the sensor is attached. Therefore, the *object reset* function has been applied to Xsens MTi-9 sensor by using the related SDK in Matlab before each measurement in order to control the *drift-error* of the related sensor.
  The calibration pattern is assumed to be vertical at its initial position. Since an *object reset* function has been applied to Xsens MTi-9 sensor at initial position of the calibration pattern, the values of initial rotations of the calibration pattern are equal to zero.
  The global coordinates of the corners of calibration grid-pattern at its initial position have been manually detected according to the global *origin* illustrated in Figure 1. The global origin is static in each image and the checkerboard points are fixed relative to the static global origin. The sizes of the grids on the calibration pattern are equal to 30-by-30 mm. The corners of the calibration grid-pattern have the value of *Y* = 0 at the initial position of the calibration grid-pattern. The corresponding global coordinates (*X, Y, Z*) of (*u_left_, v_left_, u_right_, v_right_*) have been computed by multiplying the related spatial coordinates of corners with the related rotation matrix.
  Xsens MTi-9 is affected by *excessive shocks, violent handling, magnetic fields* and *thermal-effects*. Therefore, all the sensor measurements have been realized by using the default *Kalman filter* of SDK of the related sensor, in order to suppress the effects of the mentioned noise sources over measurements.
  The *Harris Corner Detection* operator [30] has been used to extract the image coordinates of the corners of calibration grid-pattern as Harris-points. The *feature correspondence* problem has been solved by computing the homography of stereo images (Figure 2) using the related Harris-points (Figure 2 a,b) and Ransac algorithm [31]. Since the stereo matching problem could be solved efficiently, epipolar lines have been extracted successfully before image-matching operations (as in Figure 2 c,d). The normalized cross correlation operator has been applied to stereo images, in order to obtain image coordinates of corners (*u_left_, v_left_, u_right_, v_right_*) of calibration grid-pattern, where (*u_left_, v_left_*), (*u_right_, v_right_*) denotes the image coordinates of the related corners in the left and right stereo images, respectively.
2. *Training of RBF*: The proposed method uses a 3-layered RBF neural network in order to map 2-by-2D image coordinates to 3D global coordinates. The input layer of RBF has four-inputs for the left and right image coordinates (*u_left_, v_left_, u_right_, v_right_*) of the observed point; and the output layer of RBF has three-neurons that correspond to the global coordinates of the related point. The RBF has been trained as explained in Section 2.1. The test error of RMS-fitness function is 0.017 and has been computed as seen in Table 1.
3. *3D Reconstruction of Test Object*: In this step, a predefined texture pattern has been projected onto the test object (i.e., The face of Author) located inside the motion-volume of rotating-plane by using a DLP data projector. The stereo image coordinates of the projected texture pattern have been acquired from the rectified images by using normalized cross correlation. The obtained stereo image coordinates have been applied to the trained-RBF, in order to compute the corresponding global coordinates, [*X Y Z*]*_test_*.
  The noises within the computed global coordinates, which are affected from image-matching errors, have been eliminated by using the FastRBF toolbox [32].
  Surface meshing and mesh smoothing have been intensively used in 3D visualization applications. The FastRBF toolbox offers several techniques for fitting radial based functions to measured data including error-bar fitting, spline smoothing and linear filtering. In this paper, the linear filtering technique of RBF has been used. FastRBFs implicit surface meshing and mesh smoothing tools are particularly useful for reconstructing surfaces from point cloud range data. Therefore, the FastRBF tools have been used in mesh generation and smoothing of the author's face, illustrated in Figure 2(e).

### 3.1. Modified Direct Linear Transformation

The Direct Linear Transformation (DLT) [24] has been commonly used for 3D data acquisition in computer vision. The DLT and its derivatives are perhaps the mostly used camera calibration techniques in the computer vision literature. Therefore, the MDLT has been employed as a comparison method in this paper.

The DLT method uses a set of precalibrated-control points whose 3D global and 2D image coordinates are already known. The control points have been fixed to a physical object, known as the calibration object. The DLT equations consist of 11 parameters even though the system has only 10 independent unknown factors since the *principal distance* and the *scale factor*s are mutually dependent. Therefore, a non-linear constraint has been added to DLT by MDLT.

The MDLT is defined as;

(3) $$u=\frac{L_{1}X+L_{2}Y+L_{3}Z+L_{4}}{L_{9}X+L_{10}Y+L_{11}Z+1}$$

(4) $$v=\frac{L_{5}X+L_{6}Y+L_{7}Z+L_{8}}{L_{9}X+L_{10}Y+L_{11}Z+1}$$

where (*u,v*) and *L_i_* (*i* = 1, 2, 3, …, 11) denote the image coordinates and the coefficients of DLT, respectively. The non-linear constraint of the MDLT is defined as,

(5) $$\frac{L_{1}L_{5}+L_{2}L_{6}+L_{3}L_{7}}{L_{1}L_{9}+L_{2}L_{10}+L_{3}L_{11}}=\frac{L_{5}L_{9}+L_{6}L_{10}+L_{7}L_{11}}{L_{9}^{2}+L_{10}^{2}+L_{11}^{2}}$$

The *geometrical distortions* of image coordinates have been eliminated by using the related camera parameters, which has been explained in the next subsection, in order to increase the success of the MDLT. In this paper, a manually calibrated 3D object has been used in order to calibrate the MDLT. The well-known *iterative least square solution* of the MDLT has been realized in Matlab. Readers interested in the details of MDLT may refer to an excellent study on this subject [24].

### 3.2. The Camera Model

The cameras used in this paper have been calibrated by using the camera calibration toolbox given in [2]. The camera calibration parameters of the test cameras are given at Table 2. Since RBF heuristically models the geometrical mapping from 2D-to-3D, the proposed method uses only the observed image coordinates and it does not require making any correction on the observed image coordinates. The image coordinates have been geometrically corrected for MDLT by using the related camera calibration parameters given at Table 2.

Let the distorted image coordinates, (*x_i_, y_i_*), with radial and tangential lens distortions be defined as;

(6) $$\begin{array}{c} x_{u}=x_{0}-f_{x}\left[\frac{m_{11}(X-X_{0})+m_{12}(Y-Y_{0})+m_{13}(Z-Z_{0})}{m_{31}(X-X_{0})+m_{32}(Y-Y_{0})+m_{33}(Z-Z_{0})}\right]\\ y_{u}=y_{0}-f_{y}\left[\frac{m_{21}(X-X_{0})+m_{22}(Y-Y_{0})+m_{23}(Z-Z_{0})}{m_{31}(X-X_{0})+m_{32}(Y-Y_{0})+m_{33}(Z-Z_{0})}\right] \end{array}$$

where

(*x_u_, y_u_*) : lens-distorted image coordinates
(*x*_0_, *y*_0_) : image coordinates of principal point
(*f_x_, f_y_*) : focal lengths
(*m*_11_, *m*_12_,…, *m*_33_) : elements of rotation matrix
(*X*_0_, *Y*_0_, *Z*_0_) : global coordinates of projection center

The elements of rotation matrix are defined as:

(7) $$\begin{array}{l} m_{11}=\cos\phi \cos\kappa \\ m_{12}=\sin\omega \sin\phi \cos\kappa +\cos\omega \sin\kappa \\ m_{13}=-\cos\omega \sin\phi \cos\kappa +\sin\omega \sin\kappa \\ m_{21}=-\cos\phi \sin\kappa \\ m_{22}=-\sin\omega \sin\phi \sin\kappa +\cos\omega \sin\kappa \\ m_{23}=\cos\omega \sin\phi \sin\kappa +\sin\omega \cos\kappa \\ m_{31}=\sin\phi \\ m_{32}=-\sin\omega \cos\phi \\ m_{33}=\cos\omega \cos\phi \end{array}$$

where (*ω, ϕ, κ*) denote the rotation angles. The normalized point coordinates, (*x_i_, y_i_*), are defined as,

(8) $$\begin{array}{l} x_{i}=(1+k_{1}r^{2}+k_{2}r^{4}+k_{5}r^{6})x_{u}+2k_{3}x_{u}y_{u}+k_{4}(r^{2}+2x_{u}^{2})\\ y_{i}=(1+k_{1}r^{2}+k_{2}r^{4}+k_{5}r^{6})y_{u}+k_{3}(r^{2}+2y_{u}^{2})+2k_{4}x_{u}y_{u}\\ r=x_{u}^{2}+y_{u}^{2} \end{array}$$

where (*k*_1_, *k*_2_, *k*_3_,…,*k*_5_) denote the distortion coefficients (*radial* and *tangential* distortions). Thus, the final pixel coordinates, (*x_imj_, y_imj_*), on the image plane are defined as;

(9) $$\begin{array}{l} x_{\text{imj}}=f_{x}(x_{i}+S_{x}y_{i})+x_{0}\\ y_{\text{imj}}=f_{y}y_{i}+y_{0} \end{array}$$

where *S_x_* denotes *skew coefficient* defining the angle between the axes of the pixel [2]. The Equations 6-9 explain the mathematical model of the image acquisition system.

### 3.3. Xsens MTi-9 Inertial Sensor

Because the camera systems suffer from several noise sources, the rotation matrix of the rotated calibration pattern has been acquired by using an MTi-9 inertial sensor. The MTi-9 is a miniature, gyro-enhanced Attitude and Heading Reference System [20, 21]. The internal processor of the MTi-9 provides error-drift free 3D orientation, 3D acceleration, 3D rate-of-turn and 3D earth-magnetic field values at 100Hz. The MTi-9 is an excellent measurement unit for stabilization and control of cameras, calibration patterns and other equipment in computer vision [20]. The small size and low weight (35 g) of the MTi-9 makes it well-suited for capturing orientation of rotating calibration pattern. With the Xsens Software Development Kit (SDK) of MTi-9, users can integrate MTi-9 sensor in any system or application. The rotation matrix of the rotated calibration pattern has been captured by using Xsens Kalman Filter (XKF) for 3 degrees of freedom orientation. XKF uses signals of the MTi-9 for the computation of dynamic movements with no drift.

## 4. Experiments

In this paper, a set of *real images* have been used in the experiments. The proposed method has been implemented by using the image processing toolbox of Matlab, and SDK of Canon Camera Control. The images of calibration pattern have been captured by using two *static, computer-controlled* and *synchronized* Canon SX110IS 9MP cameras. Therefore, the neural structure used in the proposed method has only four inputs. All the captured images were 1,600 × 1,200 pixels sized and 24 bits/pixel. One precalibrated 3D object has been used for computing the parameters of MDLT. The interior parameters (*including distortion coefficients*) of the test cameras have been computed by using the camera calibration toolbox given in [2]. Before performing the MDLT, geometric distortion corrections have been applied to the image coordinates, in order to increase the success of the MDLT. On the other hand, no distortion corrections have been applied to the image coordinates for the proposed method.

The performance of the proposed method has been examined by scanning both a 2D test object, a 3D test object and the face of the author. The experimental results of the proposed method have been compared with the experimental results of MDLT. All the measurements have been denoised by using the FastRBF toolbox [32] before performance analysis of backprojection of 2D and 3D test objects, in order to analyze effectiveness of smoothing of FastRBF.

*Planimetric* and *depth reconstruction* accuracies of the mentioned methods have been evaluated in Mean-Squared-Error (MSE) as seen at Table 3.

The experimental results verify the success of the proposed method and MDLT. All of the errors have been measured with respect to checkerboard-test object. The test points have been marked at the corners of the test object. The checkerboard-test pattern has been designed in Matlab and printed with a 9,600 DPI professional plotter and attached onto a flat board. Since 373 points/mm have been defined on test pattern for 9,600 DPI, 0.002mm (1/373mm) has been accepted as the ground truth of test object.

Totally 1,127 3D points have been captured over the 2D test pattern and totally 1,460 3D points have been captured over the author's face. All of the measurements in the global coordinates have been performed in centimeters. The solid model of the author's face obtained by using the proposed method has been illustrated in Figure 2(e). Extensive simulations show that the results of the proposed method are close to MDLT for 2D test object but they are better in both planimetric and depth perception.

**Figure 3.**
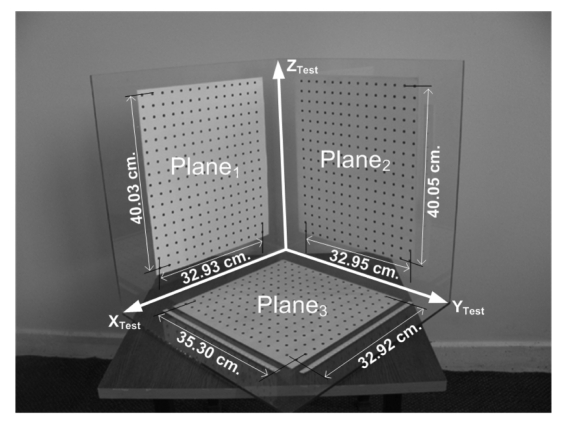
The Test Object used for performance measurement of the mentioned methods.

The 3D backprojection tests have been realized on a 3D test object, which is illustrated in Figure 3. The FastRBF toolbox based denoising phase has not been employed in 3D backprojection tests of 3D object. The related 3D test object has been located inside of the calibration volume, and its images were captured by using the cameras. The distances of 686 backprojected 3D points to the computed-planes (Figure 3) have been analyzed. The mean (*μ*) and standard deviation (*σ*) values of related distances have been computed. For the MDLT, *μ* = 2.487 mm and *σ* = 0.868 mm have been computed. For the proposed method, *μ* = 2.128 mm and *σ* = 0.793 mm have been computed. The edge lengths, illustrated in the Figure 3, have been computed by using the mentioned methods and results have been compared with the mean of the manually measured values. The manual measurements have been realized by using a vernier-caliper at the resolution of 0.01 mm. All the manual measurements of vernier-caliper have been repeated 20 times, in order to avoid reading errors of user. In Table 4, the test results on the 3D test object have been given.

## 5. Results and Discussion

In this paper, an Xsens MTi-9 inertial sensor and an RBF have been used together for 3D information recovery from images. The obtained results have been compared with the results obtained from a traditional camera calibration method, MDLT.

The main advantages of the proposed method are as follows: It does not require the knowledge of complex mathematical models of view-geometry and an initial estimation of camera calibration, it can be used with various cameras by producing correct outputs, and it can be used in dynamical systems to recognize the position of the camera after training the ANN structure. Therefore, the proposed method is more flexible and straightforward than many of the methods introduced in the literature.

The advantages of the proposed method may be summarized as follows:

- The proposed method introduces a novel implicit camera calibration method based on inertial sensors (Implicit camera calibration techniques are not interested in the physical parameters of the cameras).
- The results of the proposed method are close to MDLT but they are better, therefore it can be used in robotic vision as MDLT.
- The computational-burden of the proposed method is less than MDLT.
- The required time for preparation and scaling of the 2D calibration object of the proposed method is less than the time of preparation and scaling of the 3D calibration object of MDLT.
- It offers high accuracy both in planimetric (x,y) and in depth (z).
- It is simple to apply and fast after training.
- The image distortion and the physical parameters of the cameras have been covered by the neural network model of the proposed method.
- No image distortion model is required.
- It does not use physical parameters of cameras.
- An approximated solution for initial step of camera calibration is not employed.
- Optimization algorithms are not employed during 3D reconstruction in contrary to some of the well-known 3D acquisition methods.

## Figures and Tables

**Table 1.** Influence of several fitness functions on RBF structure.

Fitness Function	Equation	Test Error	*N* of Equation 1
RMS	$\sqrt{\frac{1}{N_{t}}\sum_{n=1}^{N_{t}}{\left(p_{n}-y_{n}\right)}^{2}}$	0.017	24
MSE	$\frac{1}{N_{t}}\sum_{n=1}^{N_{t}}{\left(p_{n}-y_{n}\right)}^{2}$	0.019	25
SSE	$\sum_{n=1}^{N_{t}}{\left(p_{n}-y_{n}\right)}^{2}$	0.021	25
MAE	$\frac{1}{N_{t}}\sum_{n=1}^{N_{t}}\left|p_{n}-y_{n}\right|$	0.033	28

**Table 2.** The camera calibration parameters of the test cameras.

Parameter	Camera #1	Camera #2

*f_x_*	1649.149	1650.865
*f_y_*	1655.941	1658.082
*x*_0_	789.515	794.803
*y*_0_	582.668	581.167
*S_x_*	0.000	0.000
*k*_1_	-0.210	-0.211
*k*_2_	0.175	0.186
*k*_3_	-0.001	-0.002
*k*_4_	0.000	0.000
*k*_5_	0.000	0.000

**Table 3.** The MSE values of backprojection of the 2D test object.

Method	MSE		
	X (cm)	Y (cm)	Z (cm)
Proposed (with original measurements)	0.07104	0.25692	0.09343
MDLT (with original measurements)	0.08193	0.34160	0.11131
Proposed (with denoised measurements)	0.00085	0.00495	0.00109
MDLT (with denoised measurements)	0.00193	0.01028	0.00277

**Table 4.** Results on the 3D test object.

	Reference Measurements		MDLT		Proposed Method	
Plane No	Edge #1	Edge #2	Edge #1	Edge #2	Edge #1	Edge #2
1	40.03	32.93	39.9336	32.9682	40.0277	32.9519
2	40.05	32.95	40.0378	32.9208	40.0239	32.9591
3	35.30	32.92	35.3193	32.9670	35.2859	32.9557
